# Bibliometric Analysis of Charcot Arthropathy (1995–2025): Current Status and Emerging Trends

**DOI:** 10.1002/jfa2.70161

**Published:** 2026-05-06

**Authors:** Jian Lin Zhou, Hao Peng, Fei Sun

**Affiliations:** ^1^ Renmin Hospital of Wuhan University Wuhan China

**Keywords:** bibliometric analysis, charcot arthropathy, charcot neuroarthropathy, charcot neuropathic osteoarthropathy, CiteSpace

## Abstract

**Background:**

Charcot Arthropathy (CA) is a destructive joint condition tied to neurotrophic and neuropathic processes. The clinical course is often complicated—high amputation rates and a generally poor prognosis have long made management difficult. Decades of research have brought progress in basic science and clinical care, yet a clear, data‐driven picture of the global research landscape and how it has shifted over time remains, in many ways, incomplete.

**Objective:**

This study set out to map the core components, current hotspots, and emerging frontiers in the CA literature.

**Methods:**

We focused on English‐language publications in the Web of Science (WoS) database from 1995 to 2025. Using tools such as CiteSpace, the R package bibliometrix, and GraphPad, we conducted a quantitative analysis of the countries or regions, institutions, authors, collaboration patterns, journals, cited references, and keywords associated with CA‐related publications.

**Results:**

We screened 349 papers. Annual output has generally trended upward—perhaps a sign of growing interest in the field. The United States ranked first in both total publications (*n* = 180) and citations (*n* = 3949), and sat at the center of international collaboration networks. Key authors (e.g., Dane K. Wukich) and institutions (e.g., the University of Texas System) emerged as major contributors; their dense collaborations appear to drive much of the field's progress. The Journal of Foot and Ankle Surgery published the most CA‐related papers (*n* = 47). Current research hotspots appear to center on the foot and ankle, pathogenesis, and surgical treatment. Looking at keyword trends, the field seems to be moving toward biomechanics, advanced reconstruction, and three‐dimensional imaging.

**Conclusion:**

This study is the first comprehensive mapping of CA literature over three decades. It highlights the United States' leading role and a shift toward technology in diagnosis and surgery. These findings may guide future research, collaboration, and clinical practice.

## 1 Introduction

Charcot Arthropathy (CA)—sometimes called neurogenic arthropathy—is a progressive, noninfectious condition that destroys joints [1]. What drives it? A loss of proprioception and protective pain reflexes, usually from central or peripheral sensory neuropathy [2]. Jean‐Martin Charcot first described it systematically in 1868 in patients with tabes dorsalis. The clinical picture is fairly characteristic—marked joint swelling, deformity, instability, and effusion [3, 4]. One odd feature, and a diagnostic headache, is the mismatch between how bad the joint looks and how little pain the patient reports. That mismatch often delays diagnosis [5]. Over time, the process can lead to serious joint dysfunction, severe deformity, and sometimes amputation, all of which take a heavy toll on quality of life.

In the past, tabes dorsalis and syringomyelia were the main culprits. But as disease patterns have changed, diabetic peripheral neuropathy has taken over as the leading cause. Diabetic Charcot arthropathy—which most often hits the foot and ankle (the so‐called “Charcot foot”)—poses real challenges for endocrinologists and orthopedic surgeons alike. We still do not fully understand how it develops. Two main theories dominate the discussion: the “neurotraumatic” and the “neurovascular.” The neurotraumatic theory, from Volkmann and Virchow, holds that repeated, unnoticed microtrauma in a joint that cannot feel pain sets off chronic inflammation, ligamentous laxity, and eventually damage to bone and cartilage [6, 7]. The neurovascular theory, first proposed by Charcot himself, focuses on autonomic dysfunction. The idea is that impaired sympathetic tone causes blood vessels to dilate, increasing blood flow and making osteoclasts more active. The result: bone resorption, localized osteopenia, and the classic signs—warmth and swelling [8, 9, 10].

Research worldwide has advanced our understanding of CA's pathogenesis, how to diagnose it early, and how to treat it in stages. But with so much being published, traditional narrative reviews cannot really capture the field's knowledge structure, its evolution, or its new frontiers. Something more systematic—something that can handle large volumes of data quantitatively—is needed.

That is where bibliometrics comes in. Bibliometrics gives you a quantitative way to map a field. It tracks development, picks out influential contributions, and spots emerging trends—all of which help you see the bigger picture of research dynamics [11]. We can measure various bibliographic elements: publications, authors, institutions, countries, keywords, and citation counts. We identify patterns in the field of study through keyword co‐occurrence analysis (to identify hotspots), citation analysis (to assess influence), and collaboration network analysis (to see who is collaborating with whom). Compared with qualitative reviews, bibliometrics offers a data‐driven, macro‐level view—one that is often easier to grasp when visualized [12].

Oddly enough, no one has conducted a formal bibliometric analysis of CA research. We thought it was time. A study like this could objectively show how the field's intellectual structure has taken shape over the past 30 years.

So here we are, looking at global CA research output from 1995 to 2025 using the Web of Science Core Collection. Our goal is to provide an overview of CA's current state in clinical and research work and to identify hotspots and emerging research frontiers. We hope this manuscript will serve as a useful guideline for clinicians and researchers to understand the field's overall landscape, identify priority areas, and consider future directions.

## 2 Materials and Methods

### 2.1 Literature Retrieval and Selection

**FIGURE 1:**
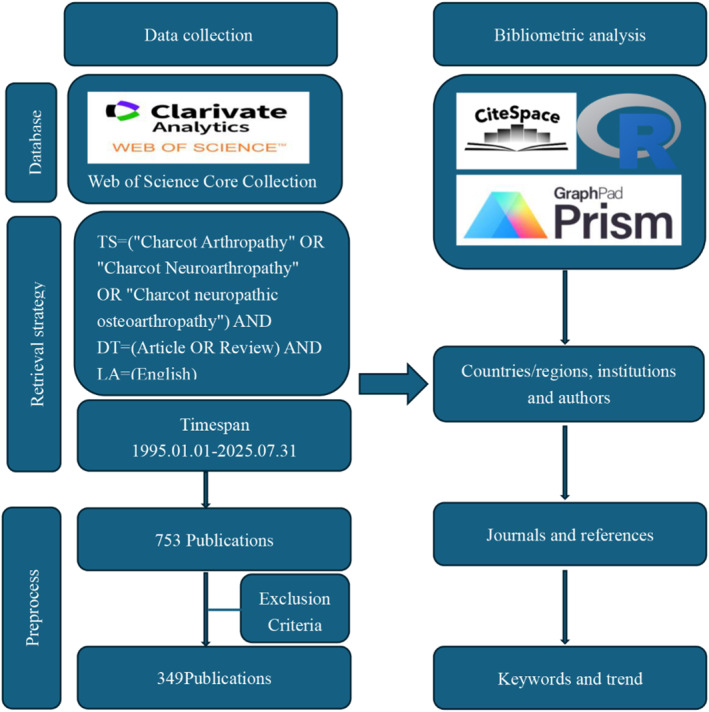
Literature search strategy and workflow of this study.

We pulled our data from the WOS Core Collection. The search criteria were: TS=(“Charcot Arthropathy” OR “Charcot Neuroarthropathy” OR “Charcot neuropathic osteoarthropathy”) AND DT = (Review OR Article) AND LA = (English). We set the time plans from January 1, 1995, to July 31, 2025. We excluded case reports, conference abstracts, editorial materials, and anything else not classified as an article or review. The initial search turned up 753 publications. After screening, we selected 349 papers for analysis. Figure 1 shows how we selected them. One thing to note: screening is never completely mechanical. For borderline cases, there is always some judgment involved—that is a limitation that comes with any literature review.

### 2.2 Bibliometric Analysis

We used CiteSpace (version 6.3.1), the R package bibliometrix (version 5.1.1), and GraphPad Prism (version 8.0). CiteSpace helped with co‐citation and co‐occurrence analyses—journals, references, keyword clusters [13]. It also lets us examine collaborative centrality for countries, institutions, and authors, and build a timeline view of merged reference clusters (handy for seeing when specific research areas emerged and how they evolved). CiteSpace also flagged keywords with strong citation bursts [14].

### 2.3 Identification of Research Frontiers

With the quantitative results from the bibliometric analysis in hand, we examined highly cited publications and emerging thematic clusters to identify current research frontiers and possible future directions in CA. This step—interpreting the data—is inherently qualitative. It reflects our reading of the field, not just numbers.

## 3 Results

### 3.1 Annual Publication Trend

**FIGURE 2:**
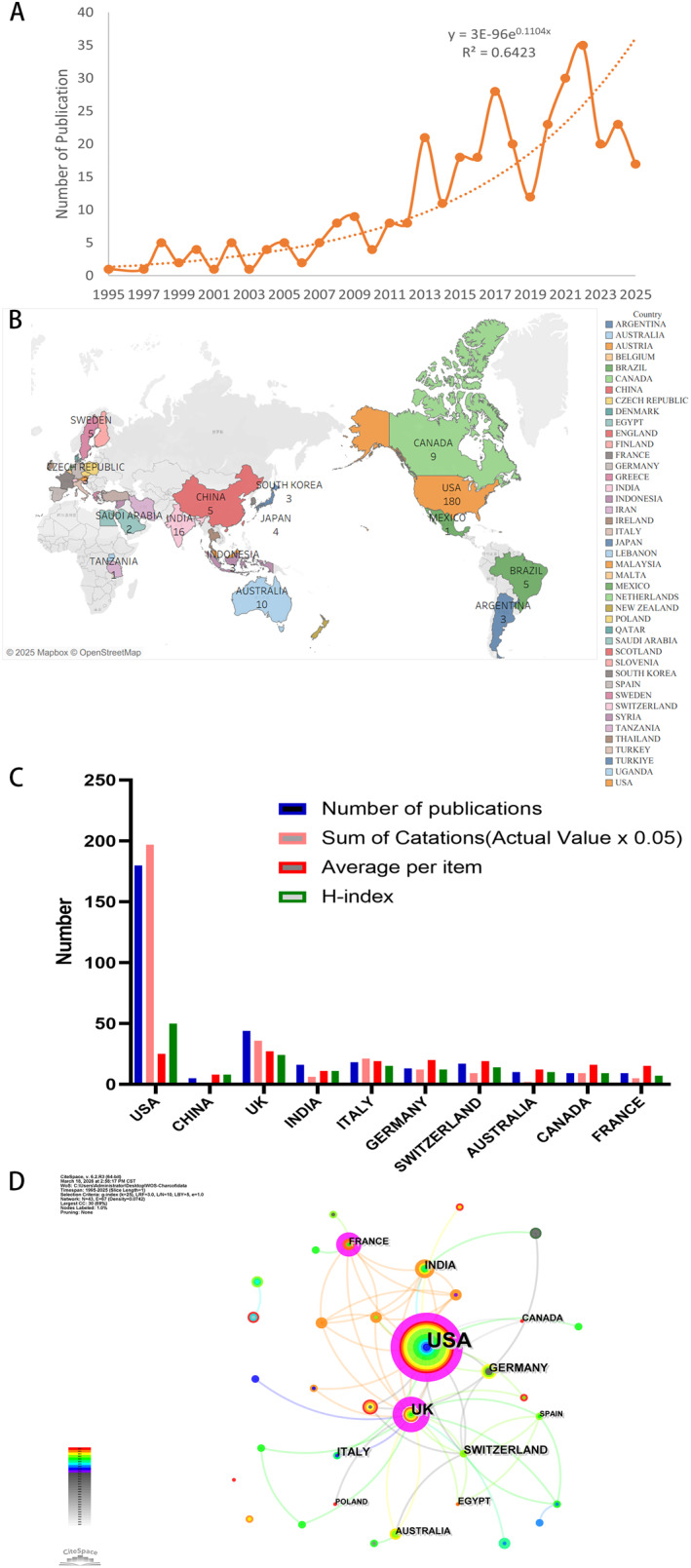
Annual publication numbers of CA, publication volume by country, citation counts, impact factors, and country collaboration network diagram. (A) Annual paper publications. (B) Distribution of publications by country. (C) Paper publications, citation counts, and H‐index of major countries compared to USA. (D) Country collaboration network diagram.

The annual number of published papers is often used as a rough indicator of research activity in a given field. We found that the trend in CA‐related publications from 1995 to 2025 showed an overall upward trajectory, although the growth was not entirely steady (Figure 2A). This trend may reflect growing interest in CA. A slight peak occurred between 2021 and 2022, with the highest number of publications recorded in 2022 (*n* = 35). It is difficult to determine whether this represents a true peak or merely a temporary fluctuation—only time will tell.

### 3.2 Analysis of Countries/Regions

Looking at publication counts by country gives a sense of where the research is happening. The United States led with 180 publications, followed by the United Kingdom with 44 (Figure 2B). US publications also showed stronger academic influence—highest total citations (3,949), high average citations, and a strong H‐index (Table 1, Figure 2C). Chronologically, the United Kingdom and the United States began earlier (in 1995 and 1998, respectively). China's first CA publication did not appear until 2015. The collaboration network map (Figure 2D) uses circle size to indicate publication volume and lines to indicate connections between countries. More lines suggest stronger cross‐country ties. The map suggests research is heavily concentrated in Western countries, with the United States as a central node. Strong links show up between the United States, United Kingdom, and France—international partnerships seem to matter. Why did China enter so late? Possibly differences in research infrastructure, funding priorities, or how common the condition is locally. Hard to know for sure.

**TABLE 1:** Ranking of country publications, publication timing, citation, and H‐index.

Country	Publications	Date of initial publication	Sum of catations	Average per item	H‐index
UNITED STATES OF AMERICA	180	1998	3949	23	50
CHINA	5	2015	15	3.8	8
UNITED KINGDOM	44	1995	735	23.7	24
INDIA	16	2013	139	11.60	11.00
ITALY	18	2005	439	25.80	15.00
GERMANY	13	2004	250	20.80	12.00
SWITZERLAND	17	2010	192	16.00	14.00
AUSTRALIA	10	2014	57	9.50	10.00
CANADA	9	2000	188	31.30	9.00
FRANCE	9	2012	5.45	15.60	7.00

### 3.3 Contributions of Institutions and Authors

**FIGURE 3:**
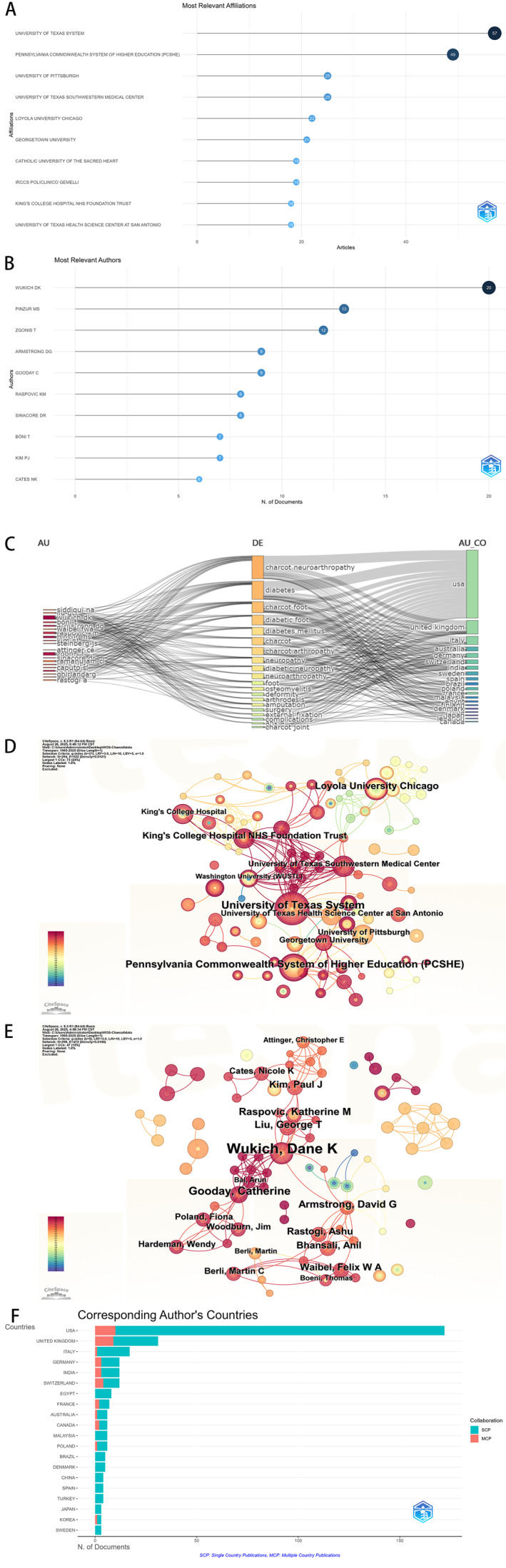
Institution and author collaboration diagram. (A) Top 10 institutions by publications. (B) Time distribution of top 10 authors. (C) Sankey diagram: relationships among authors (left), keywords (center), and author nationalities. (D) and (E). Distribution of institutional collaboration network diagram and author collaboration network diagram. (F) Author collaboration patterns.

Institutional productivity pretty much mirrors national trends. The top three institutions were all American: the University of Texas System, the Pennsylvania Commonwealth System of Higher Education (PCSHE), and the University of Pittsburgh (Figure 3A). At the author level, Wukich DK had the most publications (38) (Table 2). The timeline (Figure 3B) displays the publication records of various scholars over the past 30 years, with the size of the dots proportional to the number of papers published. Furthermore, it is evident that authors Armstrong DG and Sinacore DR (both from the United States) have consistently published research papers in CA‐related fields over the past 30 years and are early pioneers in this field. Figure 3C maps authors (left), keywords (center), and author nationalities. The rectangle size corresponds to publication volume, and the figure shows strong links among authors from the United States, United Kingdom, Italy, and elsewhere. Siddigui, NA, and Wukich DK (both U.S.) seem especially tied to the keyword “Charcot Neuroarthropathy.” The co‐authorship and collaboration networks (Figure 3D–3G) show dense, strong ties among major contributors—particularly within United States and between United States and European countries like the United Kingdom and Italy. Whether that kind of density fosters productivity or creates intellectual insularity is an open question.

**TABLE 2:** Top 10 authors by publications, countries, citation counts, and H‐index.

Rank	First author	Country	Publications	Total citations	Average per item	H‐index
1	Dane K Wukich	USA	38	1285	33.82	20
2	Thomas Zgonis	USA	20	185	9.25	8
3	Raspovic, Katherine	USA	19	418	22	12
4	Pinzur, Michael S	Canada	19	1092	57.47	15
5	Lavery, L. A	USA	14	772	55.41	12
6	Sinacore, David R	USA	14	312	22.29	9
7	Boulton, Andrew J	UK	12	1184	98.67	11
8	Jude, Edward B	UK	12	450	37.5	10
9	Ramanujam, Crystal L	USA	11	82	7.45	6
10	Armstrong, David G	USA	11	1281	116.45	9

### 3.4 Journal Sources and Co‐Cited References

**FIGURE 4:**
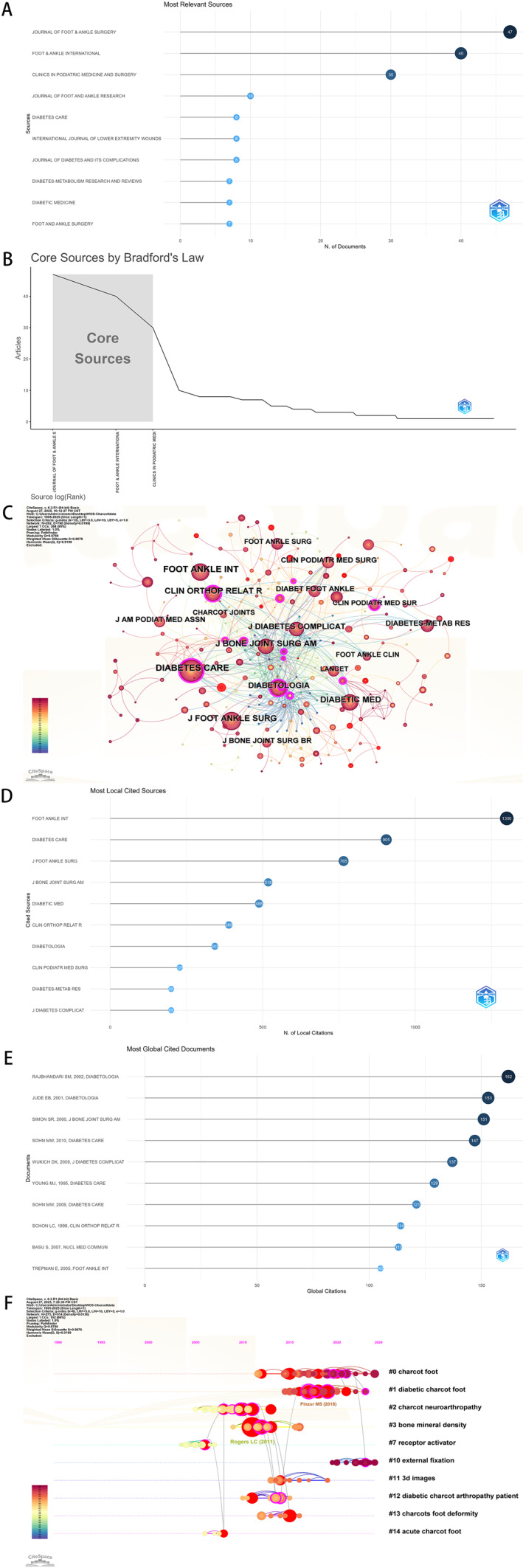
Analysis of journal sources and references. (A) Top 10 journals by publications. (B) Journals analysis by Bradford's Law. (C) Journal Co‐citation network diagram. (D) Top 10 journals by citation count. (E) Top 10 articles by citation count. (F) Timeline of E articles' total citations.

Journal sources and co‐citations can tell you something about a field's core knowledge base. Our results show that CA‐related papers were published in 127 different journals. Ranked by number of publications, the top three journals were The Journal of Foot and Ankle Surgery, Foot and Ankle International, and Clinics in Podiatric Medicine and Surgery (Figure 4A,B). According to Bradford's Law, these journals were identified as core journals. Figure 4C illustrates the co‐citation relationships among the journals; among them, Foot and Ankle International and Diabetes Care received the highest number of citations. In terms of total citations, Foot and Ankle International led (1,300), followed by Diabetes Care (905) and The Journal of Foot and Ankle Surgery (765) (Figure 4D). Co‐citation analysis of references pointed to some foundational papers: Rajbhandari SM, 2002, Diabetologia (162 citations); Jude EB, 2001, Diabetologia (153 citations); and Simon SR, 2000, Journal of Bone and Joint Surgery Am (151 citations) (Figure 4E). Timeline analysis of cited reference clusters turned up 14 distinct clusters (Figure 4F). The most recent ones—such as #0 (Charcot foot) and #11 (3D images)—might reflect current research foci. But, we should be cautious: cluster labels are algorithm‐generated and do not always capture the full nuance of the research.

### 3.5 Keyword Analysis and Research Frontiers

**FIGURE 5:**
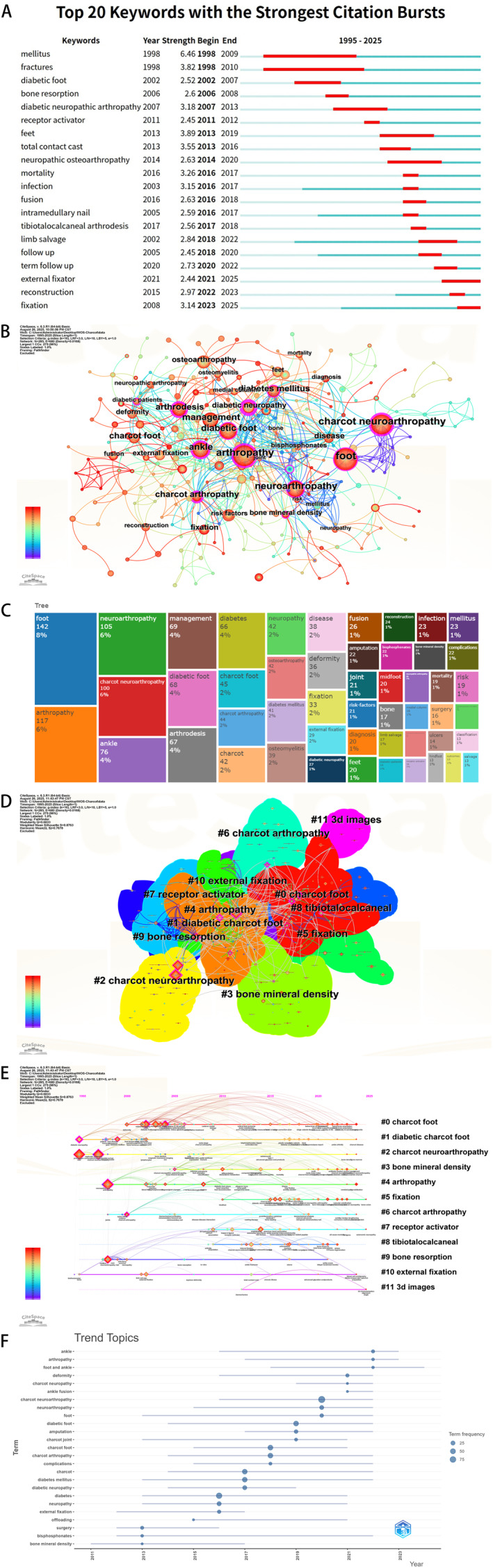
Analysis of keywords. (A) Keyword of strongest citation bursts. (B) Keyword co‐occurrence analysis. (C) Keyword co‐occurrence analysis tree dendrogram. (D) Keyword cluster analysis. (E) Keyword timeline analysis. (F) Keyword trend analysis.

Keywords give you a window into the evolution of themes and emerging trends. Figure 5A shows the top 20 keywords with the strongest citation bursts—red segments mark periods of heightened interest. The keyword co‐occurrence network (Figure 5B) shows strong thematic links; larger nodes mean higher frequency. The tree dendrogram (Figure 5C) shows possible keyword groupings and their relative frequencies. Cluster analysis grouped the keywords into 11 themes (Figure 5D). The timeline (Figure 5E) reveals the timing, duration, and interrelationships of these key terms. The keyword burst trend chart (Figure 5F) indicates that “foot,” “arthropathy,” and “neuroarthropathy” have consistently been central to the research focus of these articles. It should be noted that keyword analysis is sensitive to inconsistencies in authors' use of terminology. Inconsistent terminology may introduce bias.

## 4 Discussion

As far as we know, this is the first time someone has used bibliometrics to map the global CA research landscape across three decades. The main takeaway: both annual publications and citations have risen fairly steadily. That likely reflects growing interest—clinical and scientific—in this challenging condition. But it is also possible that part of the increase is just part of a broader trend: biomedical publishing has been growing across the board. Our study design cannot tease those two apart.

There is a clear geographic imbalance in research output. The United States leads the field in CA‐related research—it has published the most papers and received the most citations. China's contributions, by contrast, are modest, came later, and have a lower impact. Turkish researchers who did their own bibliometric analysis on Charcot foot also found that the United States accounted for the largest share of papers and citations, and that US–UK collaborations were especially strong [15]. Our findings line up with theirs. The imbalance suggests there is room for greater international engagement and investment beyond traditional Western research centers. What explains the concentration? Hard to say for sure. Differences in healthcare systems, how foot care is reimbursed, or even the underlying prevalence of diabetic complications might all play a role.

US dominance reappears at the institutional and author levels. Seven of the top 10 institutions are American; the University of Texas System is the most productive. Dane K. Wukich is the most prolific author, with high citation counts and a central spot in the co‐authorship network [16, 17]. There are clearly some well‐established, influential research teams—mostly in North America and Europe—and their dense collaborations seem to be a major engine driving the field forward. One could also argue, though, that depending heavily on a few research groups carries some risk. If their priorities shift, the field's momentum might shift too.

Journal analysis suggests the field is anchored in specialized, clinically oriented journals [18]. Bradford's Law—a standard bibliometric tool for identifying core journals [19] —pointed to The Journal of Foot and Ankle Surgery, Foot and Ankle International, and Clinics in Podiatric Medicine and Surgery. These three journals rank among the top in both the number of CA‐related papers published and the number of citations received, underscoring their authority in CA research. Citation network analysis helps map the knowledge landscape of CA‐related research and efficiently identifies the most highly cited papers, which often represent authoritative perspectives in the field [20, 21]. The most cited paper—Rajbhandari et al.’s “Charcot Neuroarthropathy in Diabetes Mellitus” [22] —along with other top‐cited papers, focuses on pathogenesis, diagnosis, and management. That is consistent with the idea that these have been long‐standing priorities. The reference clusters that emerged more recently—such as #0 (Charcot foot) and #11 (3D images)—might signal new directions. But, it is too early to know whether they represent passing fads or real shifts in the field. Future citation patterns will tell.

Keyword analysis gives us a finer‐grained view of current and emerging trends [12, 23].

The strongest citation bursts and central co‐occurrence nodes revolve around “foot,” “arthropathy,” and “neuroarthropathy”—so these remain core themes. Our findings show that foot and ankle pathology is central to CA research. Others have noted that CA mostly affects the foot and ankle [24, 25, 26], and our results are broadly in line with that [16, 27, 28]. The timeline and keyword trend views suggest that future research will mostly continue along these major clinical themes, perhaps integrating new technologies, such as biomechanics and 3D printing, to address complex challenges in CA joint reconstruction. But, there is often a gap between what technology can do and what actually gets adopted in practice—cost, access, surgeon training. The literature does not always capture those barriers.

To our knowledge, this is the first systematic review of CA using bibliometric methods. There are a few things this approach offers. First, it provides quantitative confirmation that the field has been growing. Second, it moves beyond subjective review by using keyword and reference clusters to identify research fronts. Third, it maps the social structure—who's collaborating with whom, who the key contributors are—which could be useful for thinking about future partnerships or training. And finally, the co‐citation network traces the intellectual pathways and foundational work that have shaped the field.

We should be upfront about the limitations. We searched only for CA‐related publications in WOS. Although this database is widely recognized among scholars, we may have ignored relevant literature in other databases (such as EMBASE and Scopus) or in nonEnglish sources. Additionally, our work was limited to July 2025, so recently published publications were not included. Finally, although our quantitative analysis tools are highly reliable, certain analytical steps—such as interpreting keyword clustering analysis—still require the subjective judgment of the data analyst. In the future, it would be more significant to update this study by incorporating data from a longer time span, additional high‐quality databases, and a broader range of language sources.

## 5 Conclusion

Our bibliometric analysis of CA provides a detailed account of the field's evolution and future directions. Clearly, the United States leads the field, with research increasingly shifting toward new surgical reconstruction techniques and therapeutic management. Looking ahead, fostering broader and deeper international collaboration will be crucial. This macro‐level perspective provides researchers and clinicians with a useful starting point, helping them understand current research hotspots in CA and inform future clinical practice.

## Author Contributions

**Jian lin Zhou:** writing – original draft preparation, writing – review and editing, formal analysis, investigation, methodology, resources, software. **Hao Peng:** funding acquisition, writing – review and editing, formal analysis, investigation, methodology, resources, software. **Fei Sun:** conceptualization, data curation, formal analysis, writing – review and editing, validation, project administration, supervision.

## Funding

This work was supported by the National Natural Science Foundation of China (Grant 81672154).

## Ethics Statement

The authors have nothing to report.

## Conflicts of Interest

The authors declare no conflicts of interest.

## Data Availability

The datasets used or analyzed during the current study are available from the corresponding author on reasonable request.
